# Plaque Characteristics in Young Adults With Symptomatic Intracranial Atherosclerotic Stenosis: A Preliminary Study

**DOI:** 10.3389/fneur.2022.825503

**Published:** 2022-02-10

**Authors:** Ling Li, Min Tang, Xuejiao Yan, Jie Gao, Niane Ma, Xiaorui Shi, Yaxin Niu, Yu Wen, Kai Ai, Xiaoyan Lei, Xiaoling Zhang

**Affiliations:** ^1^Department of Magnetic Resonance Imaging, Shaanxi Provincial People's Hospital, Xi'an, China; ^2^Philips Healthcare, Xi'an, China

**Keywords:** symptomatic intracranial atherosclerotic stenosis, high-resolution magnetic resonance imaging, ischemic stroke, young adult, plaque

## Abstract

**Purpose:**

To determine how intracranial vascular wall and atherosclerosis plaque characteristics differ between young and old adults with sICAS.

**Methods:**

Eighty-four consecutive patients with sICAS who underwent high-resolution magnetic resonance imaging (HRMRI) from December 2017 to July 2020 were retrospectively collected. These participants were divided into young adult group (18–50 years, *n* = 28) and old adult group (>50 years, *n* = 56). Reviewers were blinded to any clinical information and HRMRI scans were analyzed for qualitative and quantitative indicators of vascular walls and plaque at the maximal lumen narrowing site using the independent-sample *t*-test, Mann–Whitney *U*-test, chi-square test or Fisher exact test, and logistic regression analysis.

**Results:**

Young patients with sICAS had significantly smaller maximum wall thickness (1.45 ± 0.38 vs.1.75 ± 0.51 mm^2^, *P* = 0.003), higher prevalence of positive remodeling (53.57 vs. 21.43%, *P* = 0.003), and lower prevalence of diabetes mellitus (14.29 vs. 35.71%, *P* = 0.04) than old patients. Plaque burden and other plaque features were comparable between young and old patients.

**Conclusion:**

Young patients with sICAS have smaller maximum wall thickness and greater ability to reconstruct, and are more likely to show positive remodeling, which may lead to some atherosclerotic lesions being missed. Young patients with evidence of vessel narrowing should be carefully examined for presence of high-risk atherosclerotic plaque.

## Introduction

Symptomatic intracranial atherosclerotic stenosis (sICAS) is an important cause of ischemic stroke worldwide (1, 2). In the past, sICAS was generally reported in older individuals, but more and more young adults are being affected nowadays due to earlier appearance of traditional vascular risk factors (e.g., hypertension, diabetes, hyperlipidemia) as a result of rapid improvements in living standards (3, 4). In a recent large study from China, atherosclerosis accounted for 43.7% of ischemic strokes in young adults (5). In the young, the high risk of recurrence after a stroke and the many ensuing years of disability and loss of productivity places a huge burden on family, society, and the economy; therefore, sICAS in the young is receiving increasing attention (6–9). However, current guidelines from the American Heart and Stroke Association and the Royal College of Physicians still have few specific recommendations for the management of stroke in young adults (10–12).

Atherosclerosis—including the formation of the atherosclerotic plaque, artery stenosis, and rupture of unstable plaque—is an important cause of ischemic stroke. Early interventions to limit the occurrence and development of atherosclerotic plaque can help prevent end-stage cerebrovascular events (13). Traditional arterial imaging techniques (such as CTA, MRA, DSA) can reveal the degree of stenosis of the affected vessels, but cannot evaluate the characteristics of atherosclerotic plaque. High-resolution MRI (HR-MRI) is increasingly being recognized as a useful modality for evaluating the characteristics of vascular wall lesions and the morphological and quantitative characteristics of plaques (14, 15). However, due to paucity of research on the topic, it is not clear whether there are differences in the characteristics of atherosclerotic plaque between young and old adults (6, 16–18). Therefore, the aim of this study was to determine whether the characteristics of intracranial vascular wall and atherosclerotic plaque differ between young and old adults and to establish the value of HR-MRI for evaluation of atheromatous plaque.

## Materials and Methods

### Ethics

The study was approved by the Ethics Committee of our institution and was performed in accordance with the tenets of the 1964 Declaration of Helsinki and its later amendments or comparable ethical standards. Informed consent was obtained from all individual participants included in the study.

### Patients

The data of consecutive of patients with cerebrovascular symptoms who underwent HR-MRI at our hospital between December 2017 and July 2020 were collected from the hospital database and retrospectively analyzed. The inclusion criteria were (1) age 18–80 years; (2) clinical symptoms of classic transient ischemic attack (TIA) or ischemic stroke; (3) HR-MRI performed within 2 weeks of symptom onset; (4) at least one intracranial atherosclerotic plaque identified on HR-MRI; and (5) The clinical symptoms of TIA or ischemic stroke was attributed to plaques of atherosclerotic stenosis of intracranial portion. Classic TIA was defined as distinct focal neurologic dysfunction or monocular blindness lasting <24 h. Complete ischemic stroke was defined as one or more minor (non-disabling) completed strokes with persistence of symptoms or signs for more than 24 h (19). The exclusion criteria were (1) non-atherosclerotic intracranial artery stenosis (e.g., due to Moyamoya disease, arterial dissection, vasculitis, reversible cerebral vasoconstriction syndrome, antiphospholipid antibody syndrome, or hematological disorders.); 2) extracranial carotid artery stenosis ≥50%; (3) cardiogenic stroke; (4) incomplete clinical information or laboratory results; and (5) poor image quality. The 84 patients that met these criteria were divided into two groups according to age: a young group (18–50 years old; *n* = 28) and an old group (9) (≥50 years old; *n* = 56).

### MRI Protocol

On admission, all patients underwent MRI (DWI, T1WI, T2WI, and FLAIR) on a 3.0T MR scanner (Ingenia CX, Philips Healthcare, The Netherlands) with a 32-channel neurovascular coil. HR-MRI scan was performed within 2 weeks of symptom onset. Scan included TOF-MRA angiography, BB-T1WI imaging, BB-T2WI imaging, PDWI Vista imaging, and postcontrast T1WI. The total scanning time was about 50 min. Table 1 lists the MRI sequences and parameters.

**Table 1:** Imaging parameters for each sequence.

**Imaging parameters**	**TR (ms)**	**TE (ms)**	**FOV (mm)**	**Matrix (mm)**	**Slice thickness (mm)**	**Slice gap (mm)**
T1WI	2,000	20	230 × 180	480 × 480	6	1
T2WI	2,500	80	230 × 180	480 × 480	6	1
FLAIR	6,000	120	230 × 180	480 × 480	6	1
DWI	2,562	94	230 × 230	224 × 224	6	1
TOF-MRA	18	3	180 × 180	256 × 180	0.5	0
BB-T1WI	700	14	80 × 80	256 × 256	1.0	0.5
BB-T2WI	2,500	67	80 × 80	256 × 256	1.0	0.5
PDWI	2,400	17	80 × 80	256 × 256	1.0	0.5

*TR, repetition time; TE, echo time; FOV, field of view*.

*Postcontrast T1WI was performed 5 min after the injection of a single dose (0.1 mmol/kg of body weight) of gadolinium-based contrast agent (Magnevist; Bayer HealthCare Pharmaceuticals)*.

All MRI images were transformed to semi-automatic software (tsimaging.net) and RadiAnt DICOM Viewer (version 2020.2; http://www.radiantviewer.com) for analysis. Morphology and quantitative index of intracranial plaque at the site of maximum lumen narrowing (MLN) were analyzed. First, multiplanar reformations tool in postprocessing software was used to reconstruct the postcontrast T1WI images in both long and short axes, according to the vascular orientation at the MLN site. Traditional measurements and analysis were performed on a workstation by two experienced neuroradiologists (with 12 and 6 years of experience, respectively) who were blinded to the clinical data. The inner lumen and outer wall were manually outlined at the MLN site on reconstructed postcontrast T1WI images of each patient. The reference site was the nearest plaque-free segments proximal or distal to the MLN site. Maximum wall thickness (MWT), total vessel area (TVA), and lumen area (LA) were each measured thrice, and the values were averaged. Then, wall area (WA), plaque area (PA), plaque burden (PB), degree of stenosis (DS), remodeling index (RI), and remodeling type were calculated using the following formulas:


$$\begin{array}{lll} \text{WA} & = & \text{TVA}-\text{ LA}\\ \text{ PA} & = & {\text{WA}}_{\text{MLN}}-WA_{\text{reference}}\\ \text{ PB} & = & \left(1-\text{LA}/\text{TVA}\right)\times \;100\%\\ \text{ DS} & = & \left(1-{\text{LA}}_{\text{MLN}}/{\text{LA}}_{\text{reference}}\right)\;\times 100\%\\ \text{ RI} & = & {\text{TVA}}_{\text{MLN}}/{\text{TVA}}_{\text{reference}} \end{array}$$

RI ≥ 1.05 was considered as positive remodeling (PR), and RI < 1.05 as non-positive remodeling (20–23).

Intraplaque hemorrhage (IPH) was considered to be present if the T1WI signal within the plaque was ≥150% of the T1WI signal of adjacent muscle or pons (24). Eccentric stenosis was considered present if the thinnest part of the wall was <50% of the thickest point on at least one T1WI slice. Concentric stenosis was diagnosed if the thinnest part of the wall was estimated to be no <50% of the thickest point on all image slices or a stenosis without wall thickening (22). Plaque enhancement on postcontrast T1WI was classified as “strong” if enhancement was equal to that of pituitary parenchymal enhancement or as “not strong” if enhancement was less than that of pituitary parenchymal enhancement (25). The irregular plaque surface was defined as the surface of uneven fluctuation (26) or ulceration that was identified on multicontrast MR vessel wall images with published criteria (27). Atherosclerotic plaque in the middle cerebral artery was defined as anterior circulation plaque and in the basal artery as posterior circulation plaque. The qualitative characteristics of intracranial arterial plaque were determined independently by two neuroradiologists who were blinded to the clinical data; disagreements were resolved by consensus.

### Clinical Data Collection

Data were collected on age; sex; smoker; hypertension (systolic blood pressure ≥140 mm Hg and/or diastolic blood pressure ≥90 mm Hg); diabetes mellitus [fasting glucose ≥7.0 mmol/L, random glucose ≥11.1 mmol/L, or hemoglobin A1c (HbA1c) ≥7%]; HbA1c; hyperlipidemia [total cholesterol ≥5.18 mmol/L, triglycerides ≥1.7 mmol/L, low-density lipoprotein cholesterol (LDL) ≥ 3.37 mmol/L, high-density lipoprotein cholesterol (HDL) ≤1.04 mmol/L]; hyperhomocysteinemia (homocysteine ≥15 μmol/L); history of coronary artery disease; and family history of cardiovascular disease.

### Statistical Analysis

Intraclass correlation coefficient (ICC) was used to evaluate interobserver agreement in measurement of MWT, TVA, and LA, and was classified as very good (*r* = 0.81–1.00), good (*r* = 0.61–0.80), moderate (*r* = 0.41–0.60), fair (*r* = 0.21–0.40), or poor (*r* < 0.20).

Normally distributed continuous variables were expressed as means ± standard deviation and non-normally distributed continuous variables as medians (25th−75th percentiles); comparison between groups was performed using the independent sample *t-*test or the Mann–Whitney *U*-test, respectively. Categorical variables were summarized as counts and percentages and compared using the chi-square or Fisher exact test. Logistic regression (binary variable) analysis was performed to assess the differences in plaque features between young and old adults after adjusting for confounding factors, and the results were expressed by the regression slope (β) or odds ratio (OR) and corresponding 95% confidence intervals (CIs). Clinical risk factors were considered to be potential confounders when the *P*-value was <0.1 during comparison analysis between young and old adult. For all other tests, statistical significance was at *P* < 0.05. Statistical analysis was performed using SPSS 25.0 (IBM Corp., Armonk, NY, USA).

## Results

### Demographic Data

**Figure 1:**
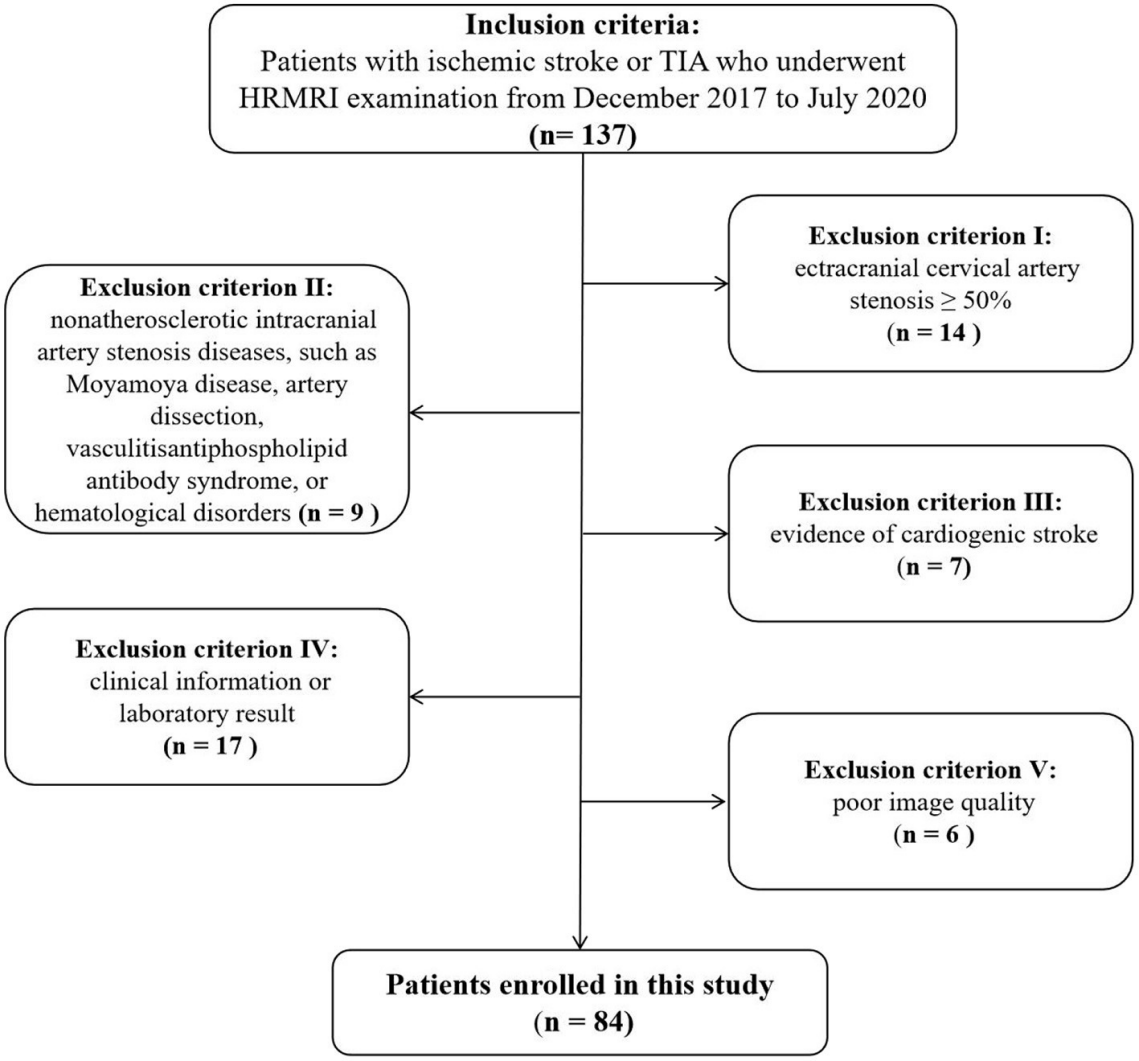
Flow chart showing process of study population selection.

The flow diagram in Figure 1 summarizes the patient selection process. A total of 84 patients (70 men; mean age, 56.6 ± 16.1 years) with sICAS were included in this study. While 51 (60.71%) patients had complete ischemic stroke (on DWI), the other 33 (39.28%) had TIA. The plaque was in the anterior circulation in 38 (45.24%) patients, and in the posterior circulation in 46 (54.76%) patients.

Of the 84 patients, 28 (33.33%) were assigned to the young adult group (22 men; mean age, 38 ± 8 years) and 56 (66.67%) patients to the old adult group (29 men; mean age, 64 ± 9 years). Table 2 shows the characteristics of the two groups. The young group had significantly lower prevalence of hypertension (53.57 vs. 78.57%, *P* = 0.018) and diabetes mellitus (14.29 vs. 35.71%, *P* = 0.04); significantly lower mean systolic blood pressure (135.25 ± 19.86 mmHg vs. 147.57 ± 25.83 mmHg, *P* = 0.038) and HbA1c (5.96 ± 1.52% vs. 6.17 ± 1.17%, *P* = 0.023); significantly higher prevalence of hyperhomocysteinemia (67.86 vs. 42.86%, *P* = 0.031); and significantly higher mean homocysteine level (31.28 ± 23.73 μmol/L vs. 16.90 ± 6.38 μmol/L, *P* = 0.023). Prevalence of smoking, hyperlipidemia, and coronary artery disease, were similar in the two groups; mean diastolic blood pressure was also comparable between the two groups.

**Table 2:** Characteristics of patients in the two groups.

**Characteristics**	**Young group (18–50 years,** ***n*** **= 28)**	**Old group (>50 years,** ***n*** **= 56)**	***P***
Age (y)	39.07 ± 8.61	66.02 ± 8.96	<0.001
Males	24 (85.71)	46 (82.14)	0.679
Smoker	17 (60.71)	28 (50)	0.353
Hypertension	15 (53.57)	44 (78.57)	0.018
Systolic blood pressure (mmHg)	135.25 ± 19.86	147.57 ± 25.83	0.038
Diastolic blood pressure (mmHg)	85.21 ± 13.64	86.02 ± 15.79	0.962
Diabetes mellitus	4 (14.29)	20 (35.71)	0.040
HbA1c	5.96 ± 1.52	6.17 ± 1.17	0.023
Hyperlipemia	6 (21.43)	12 (21.43)	1.000
TC (mmol/L)	3.76 ± 1.3	3.63 ± 0.95	0.439
TG (mmol/L)	1.88 ± 1.38	1.34 ± 0.65	0.127
LDL (mmol/L)	2.02 ± 0.73	1.97 ± 0.99	0.510
HDL (mmol/L)	0.99 ± 0.23	1.08 ± 0.31	0.129
Hyperhomocysteinemia	19 (67.86)	24 (42.86)	0.031
Homocysteine (μmol/L)	31.28 ± 23.73	16.90 ± 6.38	0.023
History of coronary artery disease	3 (10.71)	8 (14.29)	0.744

*Data are means ± standard deviation or n (%). P < 0.05 indicates significant difference*.

*HbA1c, glycosylated hemoglobin; TC, total cholesterol; HDL, high-density lipoprotein cholesterol; LDL, low-density lipoprotein cholesterol; TG, triglyceride*.

### Plaque Characteristics

**Figure 2:**
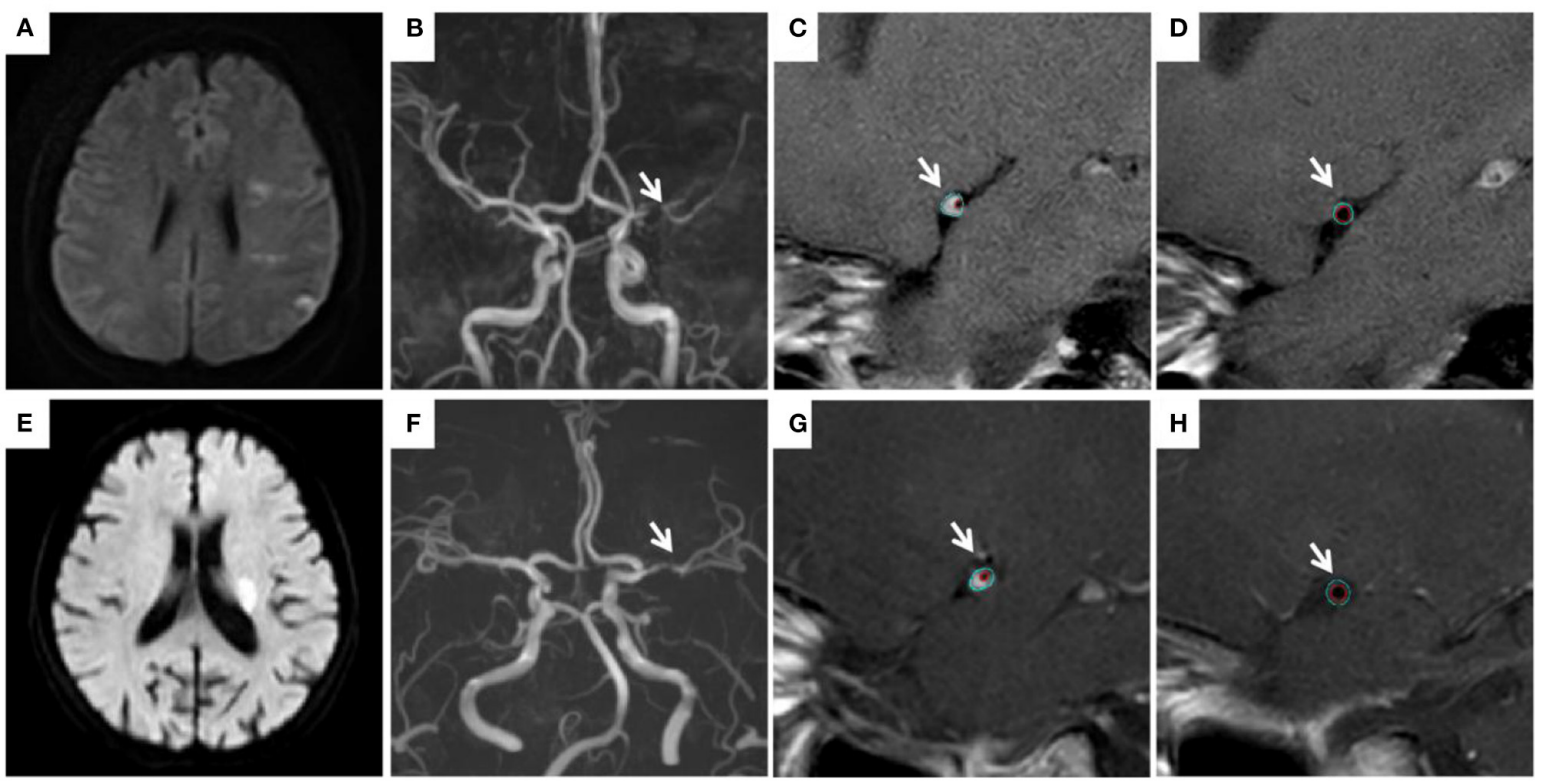
Measurement of vessel plaque characteristics. In a young patient (31 years old) with an infarction in the left hemisphere **(A)**, left MCA stenosis **(B)** was observed. TVA was 7.81 mm^2^ at the MLN site **(C)** vs. 7.10 mm^2^ at the reference site **(D)**. RI is 1.1. In a old patient (68 years old) with an infarction in the left basal ganglia **(E)**, left MCA stenosis **(F)** was observed. TVA was 7.46 mm^2^ at the MLN site **(G)** vs. 8.12 mm^2^ at the reference site **(H)**. RI is 0.92. MCA, middle cerebral artery; MLN, maximum lumen narrowing; TVA, total vessel area; RI, remodeling index.

Figure 2 shows measurement of vessel plaque characteristics. Interobserver agreement in measurements of MWT, TVA, and LA was very good, with r values of 0.969 (95% CI, 0.952–980), 0.989 (95% CI, 0.982–0.993), and 0.919 (95% CI, 0.877–0.947), respectively. Table 3 presents a comparison of plaque characteristics in the two groups. Mean MWT (1.45 ± 0.38 mm^2^ vs.1.75 ± 0.51 mm^2^, *P* < 0.003), TVA (12.0 ± 8.65 mm^2^ vs. 14.28 ± 7.08 mm^2^, *P* < 0.029), and WA (9.04 ± 5.31 mm^2^ vs. 11.41 ± 5.95 mm^2^, *P* < 0.034) were significantly lower in the young group than in the old group. Prevalence of PR was significantly higher in the young group (53.57 vs. 21.43%, *P* < 0.003). Prevalence of IPH was significantly lower in the young group (21.43 vs. 46.43%, *P* < 0.026).

**Table 3:** Plaque features in the two groups.

**Plaque features**	**Young group** **(***n*** = 28)**	**Old group** **(***n*** = 56)**	***P***
MWT (mm)	1.45 ± 0.38	1.75 ± 0.51	0.003
TVA (mm^2^)	12.0 ± 8.65	14.28 ± 7.08	0.029
LA (mm^2^)	2.96 ± 5.09	2.88 ± 2.50	0.194
WA (mm^2^)	9.04 ± 5.31	11.41 ± 5.95	0.034
PA (mm^2^)	4.36 ± 3.57	4.59 ± 3.31	0.715
PB (%)	39.04 ± 18.43	32.43 ± 17.49	0.163
DS (%)	60.14 ± 27.97	59.09 ± 25.29	0.680
RI	0.98 ± 0.41	0.79 ± 0.38	0.042
**Plaque morphology**			
PR	15 (53.57)	12 (21.43)	0.003
IPH	6 (21.43)	26 (46.43)	0.026
Strong enhancement	17 (60.71)	38 (67.86)	0.516
Surface irregularity	17 (60.71)	36 (64.28)	0.749
Eccentric distribution	17 (60.71)	42 (75)	0.177
Anterior circulation	15 (53.57)	23 (41.07)	0.278

*Data are means ± standard deviation or n (%). P <0.05 indicates a significant difference*.

*MWT, maximum wall thickness; TVA, total vessel area; LA, lumen area; WA, wall area; PA, plaque area; PB, plaque burden; DS, degree of stenosis; RI, remodeling index; IPH, intraplaque hemorrhage*.

Multivariable regression analysis revealed that MWT, PR, and prevalence of diabetes remained significantly different between young and old adult groups after adjusting for clinical confounding factors (both *P* < 0.05). MWT remained significantly lower (OR = 0.38; 95% CI: 0.002–0.635; *P* = 0.023), the prevalence of diabetes mellitus also remained significantly lower (OR = 0.173; 95% CI: 0.034–0.889; *P* = 0.036), while PR was significantly higher (OR = 5.416; 95% CI: 1.480–19.829; *P* = 0.011) in young group compared with old group. Prevalence of IPH (*P* = 0.102), hypertension (*P* = 0.062), and hyperhomocysteinemia (*P* = 0.137) were comparable between the two groups (Table 4).

**Table 4:** Results of multivariable regression analysis showing factors associated with sICAS in the young individual.

	**OR**	**95% CI**	***P***
MWT	0.38	0.002–0.635	0.023
TVA	1.008	0.871–1.167	0.914
WA	1.129	0.853–1.494	0.397
PR	5.416	1.480–19.829	0.011
IPH	0.305	0.073–1.268	0.102
Hypertension	0.276	0.071–1.067	0.062
Diabetes mellitus	0.173	0.034–0.889	0.036
Hyperhomocysteinemia	2.485	0.748–8.256	0.137

*P < 0.05 indicates a significant difference*.

*OR, odds ratio; CI, confidence interval; MWT, maximum wall thickness; TVA, total vessel area; WA, wall area; PR, positive remodeling; IPH, intraplaque hemorrhage*.

## Discussion

This study compared intracranial atherosclerotic plaque characteristics (identified with HR-MRI) and clinical factors between the young and old adult patients with sICAS and found significant differences between the two groups. Young individuals with sICAS had smaller MWT and were more likely to have PR; other plaque characteristics were similar in the two groups. Among clinical factors, diabetes mellitus was significantly less likely to be present in young patients than in old patients; other clinical factors were not significantly different between the two groups.

In this study, MWT, TVA, and WA were smaller in young patients with sICAS than in old patients but, after adjusting for potential confounders in multivariable analysis, only MWT remained significantly associated with sICAS in the young. Previous studies have also reported smaller MWT in younger patients. Cogswell et al. used 3T HR-MRI to measure internal carotid wall thickness and vessel outer wall diameter in healthy participants and found that both increase significantly with age (28). A study by de Freitas et al. (29) also showed that aging is independently related to increase in carotid intima-media thickness. The increase of MWT with age may represent the progression of atherosclerosis with age. Furthermore, instability and healing constantly alternate in atherosclerotic plaque, the repeated re-endothelialization of plaque surface and fibrosis leads to increase in MWT over time (13).

In this study, we found that PR was more likely in young patients with sICAS than in old patients. This suggests that the type of remodeling may be related to the plaque formation process. The duration of atherosclerotic plaque formation will be shorter in young patients than in old patients; because plaque development will still be in the early stage, the vessel will be more inclined to PR. However, the development of vessel tends to negative remodeling with the expand of life. Our result is consistent with reports of plaque remodeling in previous studies (30). Vessel wall compliance is better in young patients (as reflected by the lower systolic blood pressure) and so, when lumen stenosis occurs, vascular remodeling occurs more readily (31). This suggests that young patients with no stenosis or only mild stenosis, but having risk factors for early onset of large artery atherosclerosis, deserve special attention as PR may mask the presence of high-risk plaque. HR-MRI can be beneficial in such patients. In addition, PR is known to be a marker of plaque vulnerability (34), and our results showed that PR rate of young patients was higher than that of old patients, indicating that such patients had a higher incidence of vulnerable plaques, a higher risk of subsequent plaque rupture, and a higher possibility of cerebrovascular events eventually. Therefore, more attention should be paid to these patients clinically.

In the present study, young patients were found to be less likely to have diabetes mellitus than old patients; mean HbA1c was also significantly lower in young patients. It may suggests thatdiabetes mellitus has less effect on plaque' s development in young groups which has sICAS. A previous meta-analysis of 23 studies found that patients with type 2 diabetes mellitus and impaired glucose tolerance had greater carotid intima-media thickness than control patients (32). Further, Sun et al. showed that patients with high HbA1c had larger plaque burden (percent wall volume, max wall thickness) (33). This is consistent with our finding that diabetes and large MWT were less likely in young patients than in old patients.

## Limitations

Our study has several limitations. First, this was a cross-sectional study with a small sample size. Second, we did not include severely disabled patients or those with severe cardiovascular disease treated with stents, pacemakers, or implantable cardioverter defibrillators; the selection bias might have affected our results. Future longitudinal studies on large samples are warranted to clarify the impact of age on plaque characteristics. Third, measurement of quantitative plaque indices is prone to subjective errors; however, we found excellent inter-reader consistency.

## Conclusions

In conclusion, young patients with sICAS have smaller MWT and a greater ability for positive remodeling. Special attention is necessary to avoid missing the presence of high-risk plaque in these patients. HR-MRI appears to be a useful and reliable tool for evaluation of plaque characteristics.

## Data Availability Statement

The original contributions presented in the study are included in the article/supplementary material, further inquiries can be directed to the corresponding author/s.

## Ethics Statement

Ethical review and approval was not required for the current study in accordance with the local legislation and institutional requirements. The patients/participants provided their written informed consent to participate in this study.

## Informed Consent

Informed consent was obtained from all individual participants included in the study.

## Author Contributions

LL and MT drafted the manuscript and designed the study. LL performed the statistical analysis. XY and JG contributed to conducting the study and revised the manuscript. MT and KA provided technical support. LL, NM, XS, YN, and YW collected the data. XZ and XL helped design the study and revised the manuscript. All authors contributed to the article and approved the submitted version.

## Funding

This work was supported by the National Natural Science Foundation of China (81270416), the Key Research and Development Program of Shaanxi Province of China (2018ZDXM-SF-038), and the Social Development Science and Technology Research Project of Shaanxi Province of China (2021SF-064).

## Conflict of Interest

KA was employed by Philips Healthcare. The remaining authors declare that the research was conducted in the absence of any commercial or financial relationships that could be construed as a potential conflict of interest.

## Publisher's Note

All claims expressed in this article are solely those of the authors and do not necessarily represent those of their affiliated organizations, or those of the publisher, the editors and the reviewers. Any product that may be evaluated in this article, or claim that may be made by its manufacturer, is not guaranteed or endorsed by the publisher.

## Abbreviations

CI: confidence interval
DS: degree of stenosis
HbA1c: glycated hemoglobin
HDL: high-density lipoprotein cholesterol
HR-MRI: high-resolution MRI
ICC: intraclass correlation coefficient
IPH: intraplaque hemorrhage
LA: lumen area
LDL: low-density lipoprotein cholesterol
MLN: maximal lumen narrowing
MWT: maximum wall thickness
OR: odds ratio
PA: plaque area
PB: plaque burden
RI: remodeling index
sICAS: symptomatic intracranial atherosclerotic stenosis
TIA: transient ischemic attack
TVA: total vessel area
WA: wall area.
